# Management of Phantom Limb Pain through Thalamotomy of the Centro-Median Nucleus

**DOI:** 10.3390/neurolint13040058

**Published:** 2021-11-10

**Authors:** Ramiro A. Pérez de la Torre, Job J. Rodríguez Hernández, Ali Al-Ramadan, Abeer Gharaibeh

**Affiliations:** 1 Department of Research, Insight Institute of Neuroscience and Neurosurgery, 4800 Saginaw St., Flint, MI 48507, USA; ali.ramadan@iinn.com; 2 Insight Research Institute, 4800 Saginaw St., Flint, MI 48507, USA; 3 Neurosurgery Department, Stereotactic and Functional Neurosurgery Division, XXIst Century National Medical Center IMSS, Avenida Cuauhtémoc 330, Col. Doctores, Delegación Cuauhtémoc, Ciudad de México 06720, Mexico; job_6017@hotmail.com

**Keywords:** centro-median nucleus, phantom limb syndrome, thalamotomy

## Abstract

Background: Phantom limb syndrome is defined as the perception of intense pain or other sensations that are secondary to a neural lesion in a limb that does not exist. It can be treated using pharmacological and surgical interventions. Most medications are prescribed to improve patients’ lives; however, the response rate is low. In this case report, we present a case of phantom limb syndrome in a 42-year-old female with a history of transradial amputation of the left thoracic limb due to an accidental compression one year before. The patient underwent placement of a deep brain stimulator at the ventral posteromedial nucleus (VPM) on the right side and removal secondary to loss of battery. The patient continued to have a burning pain throughout the limb with a sensation of still having the limb, which was subsequently diagnosed as phantom limb syndrome. After a thorough discussion with the patient, a right stereotactic centro-median thalamotomy was offered. An immediate response was reported with a reduction in pain severity on the visual analogue scale (VAS) from a value of 9–10 preoperative to a value of 2 postoperative, with no postoperative complications. Although phantom limb pain is one of the most difficult to treat conditions, centro-median thalamotomy may provide an effective stereotactic treatment procedure with adequate outcomes.

## 1. Introduction

Most amputees experience abnormal sensations at the amputation site or the site of the amputated limb [1,2,3]. These sensations are known as postamputation phenomena, which can be painful or non-painful [1,2,3]. There are several types of these abnormal sensations, including phantom limb sensation and phantom limb pain (PLP) [4]. Phantom limb sensation is characterized by the sensation of the amputated limb being present, while PLP is the feeling of pain at the site of the amputated limb [4]. Weinstein has divided the non-painful sensation into three different types [5]. These types include kinetic sensations, kinesthetic perceptions, and exteroceptive perceptions [5]. Kinetic sensation refers to the perception of movement of the amputated part, while kinesthetic perception refers to the perception of the size, shape, and position of the body part [5]. Exteroceptive perception refers to the perception of pressure, temperature, touch, and vibration [5]. The severity of PLP ranges from mild to severe, which could have a significant impact on the patient’s life [6]. 

Chronic pain states can be secondary to trauma, vascular lesions, postsurgical procedures, inflammatory conditions, and a variety of causes. Chronic and severe pain in the body regions secondary to amputation was first observed in soldiers who were injured on the battlefield by Ambroise Paré [7]. Neuropathic pain is prevalent in the general population, accounting for 6–10% of individuals [8]. The term phantom limb syndrome was coined by neurologist Silas Weir Mitchell in 1871 and refers to the sensation that an amputated limb is still attached to the body [9]. There are other phantom sensations, which can include temperature changes, such as warmth or coldness; tingling; itching; or electrical sensations [5]. Amputees may also have the feeling of movement of the phantom limb and painful sensations referring to the absent limb [10,11].

The onset time of PLP after amputation can be from days to years [2]. Most patients experience PLP within one week after amputation, with 50% of patients experiencing PLP within 24 h after amputation [2]. Due to the increased number of amputees in the United States, most of the surgical and medical attention has been focusing on treating the recovery of function and alleviation of pain [12]. The percentage of people with PLP has been reported to be 80–100% of amputees [13]. Dijkstra and colleagues found that the prevalence of phantom pain in the total amputees’ group was 72%, with around 41% of upper limb amputees and 80% of lower limb amputees experiencing the pain [14]. This pain differs from the residual pain known as stump pain. The reported incidence of stump pain, which is the pain that is felt in the remaining part after an amputation, mainly in the scar region, can be as high as 74% and, similar to phantom pain, can persist for years [2]. While stump pain is usually due to a local pathological process [5], the exact pathophysiology of PLP is still not clear [15]. There are several mechanisms that have been proposed to explain the development of PLP [15]. More recent advances suggest that the central nervous system, the peripheral nervous system, and psychological factors are all involved in the development of PLP [15,16]. 

There are various treatment options available for PLP, including pharmacological agents, spinal cord neurostimulation, physical therapy, psychological therapy, and behavioral methods [17]. Sherman and colleagues conducted a survey of current PLP treatments and demonstrated that the effectiveness of PLP treatment differs among available treatment options [17]. Moreover, a great percentage of the amputees reported ineffectiveness of most of these treatments [17].

## 2. Case Presentation

**Figure 1:**
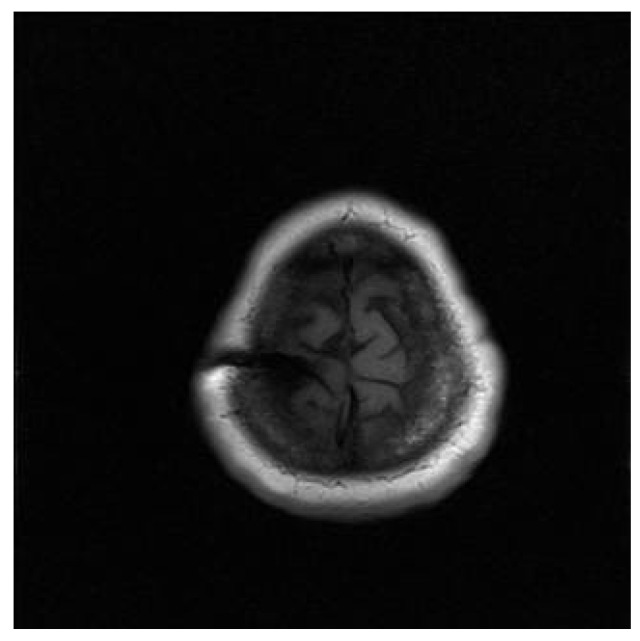
Preoperative T1 MRI sequence showing a previous brain convexity placement of an electrode at the coronal region.

This is a case of a 42-year-old female with a history of transradial amputation of the left thoracic limb due to an accidental compression one year before. The patient developed intense pain throughout the limb following the lesion, along with a burning painful sensation without any improvement with conventional and narcotic agents. The patient persisted with burning pain and a sensation of still having the limb, which was subsequently diagnosed as phantom limb syndrome. The placement of the deep brain stimulator on the right side directed to the ventral posteromedial nucleus (VPM) was performed in another center without reported complications. The initial response was adequate; however, in view of the persistence of the symptomatology, the deep brain stimulator was withdrawn. After a thorough discussion with the patient, a stereotactic centro-median right-sided thalamotomy was offered. Figure 1 shows a preoperative T1 magnetic resonance imaging (MRI) sequence showing a previous right brain convexity placement of an electrode at the coronal region.

### Surgical Technique

**Figure 2:**
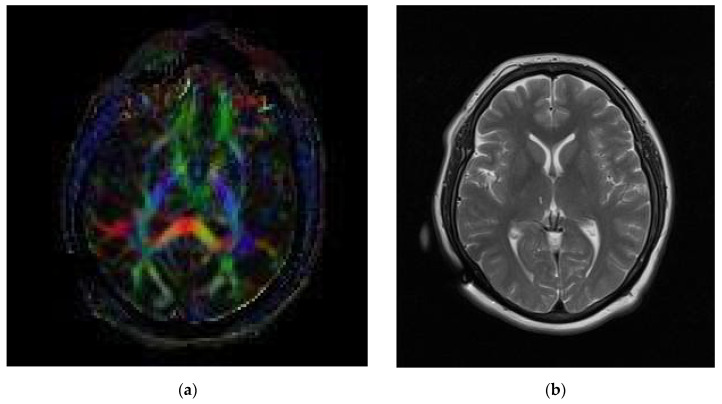
(**a**) A DTI showing tracts surrounding the left centro-median lesion. (**b**) Postoperative T2 MRI sequence showing a rounded lesion at the centro-median nucleus 6 months after the procedure.

The procedure was explained to the patient in detail, including the potential complications, such as neurodeficit, cerebrospinal fluid fistula, infection, and death. After informed consent was signed, the patient was offered a right-sided centro-median thalamotomy. A preoperative post-contrast T1 MRI sequence using 1 mm cuts through the whole brain was carried out along with a T2 MRI sequence in 1 mm cuts through the basal ganglia. Using local anesthesia, a ZD (Zamorano Dujovny, F. L. Fischer, Freiburg, Germany) frame was placed, after which, a post-contrast brain computed tomography (CT) with 1 mm cuts was carried out using the whole brain. Standard planning by means of an imaging fusion procedure was carried out (Medtronic framelink, Medtronic, Ireland) utilizing coordinates at 4–6 mm lateral to the lateral wall of the III ventricle, 8 mm post-intercommissural point, and 2–3 mm above the intercommissural line. The patient was placed under local anesthesia and sedation, and scrubbing and draping were continued in the usual fashion. A standard incision was made to reopen the prior incision, and a bilateral burr hole was made in a regular fashion to remove the prior implanted electrode and to continue the insertion of the new electrode in one side. Dural opening and coagulation were subsequently performed. A 1-mm-thick macro-electrode (Cosman, Burlington, MA, USA) with a 3 mm uninsulated tip was deepened 10 mm prior to the selected target. A macro-stimulation procedure was carried out to select the best anatomical target. When the electrode was in place at 2 mm from the target, the patient referred to touching and moving of the left arm without such maneuvers being carried out. Moreover, the patient experienced a pleasurable sensation and pain alleviation. At the target, the patient continued to have a good response to spontaneous pain, with real time and real sensation identification referred to as touching of the left arm. A coagulation procedure was subsequently performed at 70 °C for 60 s. A simple post-procedure CT was performed along with a postoperative MRI identifying the lesion location. A continuing follow-up took place for one year with adequate pain reduction from a value of 9–10 preoperative to a value of 2 postoperative on the visual analogue scale (VAS) for pain. A diffusion tensor image (DTI) shows tracts surrounding the left centro-median lesion in Figure 2a, and a postoperative T2 MRI sequence shows a rounded lesion at the centro-median nucleus 6 months after the procedure in Figure 2b.

## 3. Discussion

This case study examined the use of a thalamotomy of the centro-median nucleus for the treatment of PLP. This kind of pain is a common complaint that is experienced by amputees, and it differs from stump pain, which is the pain that is felt in the residual portion of the limb. Central nervous system mechanisms are suggested to have a role in the pathophysiology of PLP due to the structural and functional changes in the primary somatosensory cortex that occur following amputation [15,16,18]. The extent of cortical reorganization and the degree of PLP have been shown to be directly proportional [15]. Flor and colleagues showed that the degree of cortical reorganization is strongly related to the degree of reported PLP, which supports the theory that PLP is related to changes in the brain [15]. The cortical remapping theory of phantom pain or the neuromatrix theory proposes that a network of complex interactive sensory neurons connect the thalamus and the cortex as well as the cortex and the limbic system [19,20,21]. The phantom limb open-circuit theory suggests that following amputation, the return signal from that limb disappears, while the neuromatrix continues to send an outgoing signal [22]. Using different methodologies, we can apply some clinical modeling to understand the circuits that promote PLP and its interactions [23]. The afferent networks utilize all peripheral multimodal receptors, and once in the parietal lobe, they are sent back to the thalamus where it forms a typical consciousness-based circuit [23]. The loss of this loop accounts for phantom pain syndrome [23]. Peripheral nervous system mechanisms suggest that the development of PLP is due to the presence of neuromas [16]. After limb amputation, the axons of peripheral nerves are disconnected, which increases the risk of inflammation [16]. Injured nerve axons regenerate and develop neuromas [16,24,25]. These pseudotumors eventually form at the site of nerve transection [16]. The sympathetic nervous system may also be responsible for intensifying PLP, as it has been seen that the use of norepinephrine can increase the stimulation of nerve fibers [26]. In some patients, peripheral nerve blockade and anesthesia in patients with PLP were also found to attenuate or abolish PLP [26]. However, the reported beneficial effects of peripheral nerve blocks are inconsistent [27]. 

Pharmacological agents, such as gabapentin used for neuropathic pain, are the most used treatments for PLP [28,29]. However, most medical treatments are ineffective with very low response rates [22,30]. The maximum benefit that has been reported from several treatment options is about 30%, including pharmacological agents (anticonvulsants, barbiturates, neuroleptics, antidepressants, and muscle relaxants) and surgical treatments (sympathectomy, dorsal root entry zone lesions, cordotomy, rhizotomy, and neurostimulation) [22]. Peripheral nerve blockade and dorsal root ganglion blockade were found to provide a temporary relief of pain [22]. Vaso and colleagues showed that lidocaine injections given intrathecally and to the dorsal root ganglions reduced PLP symptoms [31]. 

One of the most commonly used methods to treat PLP is the use of spinal cord stimulators (SCSs), which is most beneficial for treating neuropathic pain [32,33]. Interestingly, there is also a growing trend to apply cortical stimulation to patients with PLP using cortical stimulators [34,35]. 

Psychological treatments are used as adjuvants in the treatment of PLP [36]. These psychological treatments include mirror visual feedback, hypnosis, and desensitization. Cárdenas and colleagues studied the use of psychotherapies for the treatment of phantom limb pain and concluded that more studies are needed to assess the effect of psychological therapies [36]. Barbin and colleagues showed in their systematic review that the level of evidence of the effects of mirror therapy is insufficient [37].

The use of thalamotomy for managing chronic pain conditions has been used before [38,39,40]. Franzini and colleagues studied medial thalamotomy using stereotactic radiosurgery for the treatment of intractable pain [40]. They showed that medial thalamotomy using stereotactic radiosurgery is a relatively effective and safe method [40].

The surgical treatment of PLP is difficult because of the complex etiology and expectations [2]. The management of PLP requires a comprehensive evaluation, multidisciplinary approach, and individual variations. If all pharmacological agents and narcotic usage have failed and patients continue to have excruciating pain, surgical options should be offered [41]. Although SCSs have their role in some selected patients, the cervical location necessitating a high cervical level placement along with continuous programming complicates this treatment option [42]. In this case, a centro-median thalamotomy was offered to the patient. In general, as a principle in the surgical decision, safer and simpler choices are offered initially followed by those increasing in complexity as patients complain of continuing pain. After the procedure, the patient experienced immediate pain relief that continued after one year. One finding that is considered interesting in this patient is the adequate clinical correlation between electrode placement before the target and the response of the patient along with the pleasurable sensation, which was localized 2 mm above the target. The last part of the procedure included a phantom limb sensation with the recurrence of normal cutaneous sensation on the left elbow and left arm, which has been described as a typical circuit located in the centro-median nucleus. This finding can be correlated as the reconnection of the lost loop of this circuit by means of direct stimulation. As we initially discussed, the circuits involved in phantom limb syndrome include a variety of loops. Centro-median-pf involves extensive connections with the striatal systems and the cortex. This finding is expected to stimulate additional clinical studies into the potential role this nucleus can play in a variety of clinical scenarios, including pain, Parkinson’s disease, and psychiatric conditions [43]. These descriptions, more than anatomical, are physiological in nature. However, we were able to demonstrate the existence of this circuit when the macro-electrode was positioned within the centro-median nucleus in this case. This case illustrates the importance of decision making concerning the target to be chosen. Chronic pain management continues to be challenging in some cases, allowing the operating surgeon to use all available resources within the neurosurgical armamentarium.

## 4. Conclusions

The use of stereotactic targets in pain control requires a full understanding of clinical decision making, surgical techniques, and patient’s expectations in order to select the adequate treatment for the specific clinical syndrome. Improved clinical outcomes can be offered when all these factors are considered.

## Data Availability

No new data were created or analyzed in this study. Data sharing is not applicable to this article.
